# The retina as an early biomarker of neurodegeneration in a rotenone-induced model of Parkinson’s disease: evidence for a neuroprotective effect of rosiglitazone in the eye and brain

**DOI:** 10.1186/s40478-016-0346-z

**Published:** 2016-08-18

**Authors:** Eduardo Maria Normando, Benjamin Michael Davis, Lies De Groef, Shereen Nizari, Lisa A. Turner, Nivedita Ravindran, Milena Pahlitzsch, Jonathan Brenton, Giulia Malaguarnera, Li Guo, Satyanarayana Somavarapu, Maria Francesca Cordeiro

**Affiliations:** 1 Imperial College Ophthalmology Research Group (ICORG), Department of Surgery and Cancer, Faculty of Medicine, Imperial College London, London, UK; 2 Western Eye Hospital, Imperial College Healthcare NHS Trust, London, UK; 3 UCL Institute of Ophthalmology, University College London, 11-43 Bath Street, London, EC1V 9EL UK; 4 Neural Circuit Development and Regeneration Research Group, Department of Biology, University of Leuven, Leuven, Belgium; 5 School of Pharmacy, University of London, London, UK

**Keywords:** Parkinson’s disease, Rotenone, Retina, Rosiglitazone, DARC

## Abstract

Parkinson’s Disease (PD) is the second most common neurodegenerative disease worldwide, affecting 1 % of the population over 65 years of age. Dopaminergic cell death in the substantia nigra and accumulation of Lewy bodies are the defining neuropathological hallmarks of the disease. Neuronal death and dysfunction have been reported in other central nervous system regions, including the retina. Symptoms of PD typically manifest only when more than 70 % of dopaminergic cells are lost, and the definitive diagnosis of PD can only be made histologically at post-mortem, with few biomarkers available.

In this study, a rotenone-induced rodent model of PD was employed to investigate retinal manifestations in PD and their usefulness in assessing the efficacy of a novel therapeutic intervention with a liposomal formulation of the PPAR-γ (Peroxisome proliferator-activated receptor gamma) agonist rosiglitazone.

Retinal assessment was performed using longitudinal in vivo imaging with DARC (detection of apoptosing retinal cells) and OCT (optical coherence tomography) technologies and revealed increased RGCs (Retinal Ganglion Cells) apoptosis and a transient swelling of the retinal layers at day 20 of the rotenone insult. Follow-up of this model demonstrated characteristic histological neurodegenerative changes in the substantia nigra and striatum by day 60, suggesting that retinal changes precede the “traditional” pathological manifestations of PD. The therapeutic effect of systemic administration of different formulations of rosiglitazone was then evaluated, both in the retina and the brain. Of all treatment regimen tested, sustained release administration of liposome-encapsulated rosiglitazone proved to be the most potent therapeutic strategy, as evidenced by its significant neuroprotective effect on retinal neurons at day 20, and on nigrostriatal neurons at day 60, provided convincing evidence for its potential as a treatment for PD.

Our results demonstrate significant retinal changes occurring in this model of PD. We show that rosiglitazone can efficiently protect retinal neurons from the rotenone insult, and that systemic administration of liposome-encapsulated rosiglitazone has an enhanced neuroprotective effect on the retina and CNS (Central Nervous System). To our knowledge, this is the first in vivo evidence of RGCs loss and early retinal thickness alterations in a PD model. Together, these findings suggest that retinal changes may be a good surrogate biomarker for PD, which may be used to assess new treatments both experimentally and clinically.

## Introduction

Parkinson’s Disease (PD) is the second most common neurodegenerative disease after Alzheimer’s [1], and a major cause of neurological disability in individuals over 65 years of age. PD is characterised by the loss of dopaminergic neurons in the pars compacta of the substantia nigra (SNpc) and the presence of intracytoplasmic inclusions called Lewy bodies (LBs), which consist of accumulations of both α-synuclein and ubiquitin [2]. Together, these pathological hallmarks lead to the cardinal symptoms of PD: bradykinesia, tremor, rigidity, and postural instability. LBs are also present in other regions of the central nervous system (CNS), leading to non-motor symptoms such as depression, sleep disorders, constipation, olfactory and visual dysfunction, and dementia. These are observed in 90 % of PD patients surviving 20 years [3]. Notably, Parkinsonism and PD sufferers become symptomatic only when approximately 70–80 % of dopaminergic cells are lost [4]. Hence early diagnosis and early treatment are crucial to prevent/delay disease progression, through preservation of dopaminergic cells.

Several toxin-based models for induction of PD pathology in rodents, through selective degeneration of dopaminergic cells, have been described, including administration of MPTP (1-methyl-4–phenyl-1,2,3,6-tetrahydropyridine), rotenone, or proteasome inhibitors [5]. Rotenone inhibits complex I of the mitochondrial electron transport chain, resulting in increased oxidative stress and reduced ATP synthesis, which ultimately activate mitochondria-dependent apoptosis pathways. Both pulsatile and chronic administration of rotenone in vivo and in vitro is reported to induce degeneration of nigrostriatal neurons, with formation of cytoplasmic inclusions similar to Lewy bodies, oxidative damage, parkinsonian bradykinesia and rigidity [5–8].

The occurrence of ocular manifestations in many neurodegenerative diseases, including PD, emphasizes the strong connection between the brain and the retina. Indeed, PD patients often suffer from visual symptoms such as reduced visual acuity, low contrast sensitivity and disturbed colour vision [9, 10]. In particular, recent findings have highlighted the retina as a potential biomarker for PD. Thinning of the inner retinal layer has shown to be an early event in patients with early PD, where early disease was defined as diagnosis within 2.5 years with an average disease stage of 2 (Hoehn–Yahr scale) [11, 12]. The severity of PD symptoms correlates with RNFL thickness [13, 14]. Functional changes in the retina have also been recorded in early PD (grade 1–1.5 Hoehn–Yahr scale, diagnosed within 3 years) [15] with some suggestion that RGCs are involved early [16]. Finally, using the retina as a biomarker is currently being investigated (NCT02443779, NCT02640339), and has already been used in some PD treatment trials (NCT02233023, NCT00144300). Retinal imaging technologies such as optical coherence tomography (OCT), confocal scanning laser ophthalmoscopy (cSLO), and fluorescence ophthalmoloscopy offer a non-invasive, objective opportunity to measure retinal alterations in real-time. Their application to neurodegenerative diseases in the CNS may yield novel diagnostic tests and more rapid and quantitative end-points to assess the efficacy of therapeutic interventions. One such technique is *DARC* (Detection of Apoptosing Retinal Cells), which utilizes fluorescently labelled Annexin A5 for the in vivo visualisation of apoptotic retinal ganglion cells (RGCs) [17, 18]. DARC represents a technique to quantify the extent of RGC neurodegenerative activity, which may prove useful for monitoring neurodegenerative disease onset and progression. In addition, this technique permits the investigation of the neuroprotective efficacy of promising agents in real time.

Rosiglitazone, a member of the thiazolidinedione drug family, originally used to counter insulin resistance in type 2 diabetes, has recently shown promise as a therapy in animal models of PD [19–26]. Rosiglitazone therapy is reported to promote an anti-inflammatory response, with attenuation of microglial activation, release of pro-inflammatory cytokines, oxidative stress, astrocytic gliosis and reversible inhibition of monoamine oxidase - a crucial enzyme for dopamine metabolism [19–21, 23, 24, 27]. The outcomes of clinical investigations of thiazolidinedione therapies for the treatment of PD have so far been complex. While administration of these agents is reported to not slow disease progression [28], diabetic patients receiving glitazone treatment exhibited a 28 % reduced incidence of PD [29]. One interpretation of this data is that thiazolidinedione therapy may be effective for the treatment of pre-symptomatic but not established PD, when the majority of dopaminergic cells are lost.

The aim of this study is to determine, using a rotenone-induced rodent model of PD, whether firstly, retinal imaging can identify quantifiable changes including retinal layer thickness (OCT) and RGC apoptotic counts (DARC) in the natural history of the disease; and secondly, if these changes can be used as surrogate biomarkers, and applied to assessing a potential PD treatment strategy.

## Materials and methods

### Isolation and culture of primary rat mixed retinal cultures

Rat pups aged 3 to 5 days (P3-P5) were sacrificed, and eyes removed and placed in ice-cold calcium- and magnesium-free Hank’s buffered salt solution (Thermo-Fisher). Retinas were dissected from the eye cup under a microscope, 10 retinas were pooled and treated with trypsin (0.1 % v/v; Sigma-Alrich) for 15 min at 37 °C with gentle agitation. Upon centrifugation (120 *g*, 1 min) and resuspension in Eagle’s minimum essential medium (Sigma-Aldrich), pooled retinas were subject to mechanical dissociation via gentle pipetting. Cell density was determined using a trypan blue exclusion assay (Sigma-Aldrich) and haemocytometry. Mixed retinal cultures were plated at a density of 3x10^5^ cells/well on a Poly-D-lysine (0.1 mg; Sigma-Aldrich) coated 96-well plate and cultured (37 °C, 5 % CO_2_) until 70 % confluency before use in cell viability experiments.

### In vitro cell viability assays

To determine whether rosiglitazone therapy was protective against rotenone insult, rat mixed retinal cultures were insulted for 48 h with IC_50_ concentrations of rotenone (75 nM). Rosiglitazone stock solutions were prepared by first solubilising in DMSO to a concentration of 100 mM before diluting in cell culture medium to the desired concentration. Cells were treated with 2.5 μM, 5 μM or 10 μM rosiglitazone for the second 24 h of the 48 h rotenone insult. The final concentration of DMSO was less than 0.01 % (v/v). Viability of rat mixed retinal cultures was assessed using the AlamarBlue (Invitrogen) cell viability assay according to manufacturers instructions. Briefly, 10 μl AlamarBlue solution was added to each well-plate and incubated for 2.5 h after which time the intensity of AlamarBlue fluorescence (Excitation 570 nm/Emission 585 nm) was recorded from which percentage cell viability was determined using equation 2;

$$ Viability\ \left(\%\right) = 100\left(\frac{\left(T-N\right)}{\left(H-N\right)}\right) $$

where T is the fluorescence intensity of the test well, H is the fluorescence intensity of a population of untreated (healthy) cells and N denotes a population of cells treated with a highly cytotoxic insult (0.1 % Triton-X100).

### Formulation of rosiglitazone for IP administration

Owing to the limited water solubility of rosiglitazone (LogP 2.78, ACD/LogP Freeware) for in vivo IP administration a suspension of rosiglitazone was prepared by dissolving rosiglitazone in corn oil (Fluorochem) to a concentration of 0.6 mg/ml.

To avoid the use of corn oil and DMSO, a solvent we have previous shown to induce toxicity [30], Rosiglitazone loaded liposomes were prepared using an adaptation of the lipid film hydration technique described previously [31]. Briefly, 130 mM of egg phosphatidylcholine and cholesterol (both Avanti Polar Lipids) of desired molar ratio (PC_90%_Cholesterol_10%_) were dissolved in chloroform: methanol (1:1 ratio; Sigma-Aldrich). Rosiglitazone (dissolved in the same solvent) was added to a final concentration of 3 mg/ml, before drying under a stream of oxygen-free argon until a thin film was formed. The lipid film was further dried under vacuum for 1 h, before rehydration with phosphate-buffered saline (0.01 M, pH 7.4) while warming to 45 °C with gentle mixing. The resulting multilamellar liposome solution was subject to 4 x 10 min ultrasonication and the resulting liposome suspension stabilised by incubation at 4 °C overnight. Unencapsulated rosiglitazone was removed from the liposome suspension by passing through a 0.22 μm pore filter (Sigma Aldrich) and the resulting liposome suspension was used immediately.

Spectroscopic determination of rosiglitazone concentration in liposome suspensions was achieved by diluting liposome suspensions in dimethyl sulphoxide (Sigma-Aldrich) and measuring the absorbance at 313 nm (on correcting for absorbance due to the lipid fraction), using an experimentally determined molar extinction coefficient for rosiglitazone of 4078 L.M^−1^.cm^−1^ in the same solvent system. The rosiglitazone content of liposomes was determined spectroscopically before and after 0.22 μm filtration and the percentage of incorporation determined using equation 1, where [R]_A_ is the concentration of rosiglitazone after filtration and [R]_B_ is the concentration before filtration;

$$ 100*\left(\left({\left[\mathrm{R}\right]}_{\mathrm{B}}\hbox{-} {\left[\mathrm{R}\right]}_{\mathrm{A}}\right)/{\left[\mathrm{R}\right]}_{\mathrm{B}}\right)=\mathrm{IE}\% $$

### Transmission electron microscopy

Liposomal suspensions were processed using carbon grids prior to staining with 1 % uranyl acetate solution for 1 min and drying. Observation of specimens was achieved using a transmission electron microscope (TEM) (Jeol-1010, JEOL) operated at 100 kV, with images acquired using a Orius digital camera (Gatan).

### Animal handling

All animal procedures were in compliance with the UK Home Office and with the ARVO Statement for the Use of Animals in Ophthalmic and Vision Research. Adult Dark Agouti rats (DA), 150–200 g, were acclimatised for a minimum of 7 days before the start of the treatment, and housed in a 12-h light/12-h dark cycle with unlimited access to food and water. In vivo experiments were conducted under general anaesthesia (IP) with 75 mg/kg (Ketaset; Fort Dodge Animal Health) and 0.5 mg/kg (Domitor; Pfizer).

### Rotenone-induced model of PD

A rotenone rat model of PD was induced as previously described [32]. Treatment regimens are described as below:

- Group 1 (*n* = 10): daily intraperitoneal (IP) rotenone (2.5 mg/kg in glyceryl trioctanoate, Sigma-Aldrich) administration up to 60 days (**Rot60**).
- Group 2 (*n* = 10): daily IP administration of vehicle (1 mL/kg glyceryl trioctanoate, Sigma-Aldrich) up to 60 days (**CONTROL**).
- Group 3 (*n* = 6): daily rotenone administration (see above) for 10 days, followed by a 10-days recovery period during which the animals did not receive any substances (**ROT10**).
- Group 4 (*n* = 6): daily rotenone administration for 10 days, followed by daily rosiglitazone treatment (IP 3 mg/kg in corn oil; Fluorochem) for 10 days (Rosiglitazone Intervention Group, **RIG10**).
- Group 5 (*n* = 6): daily rotenone for 10 days, followed by a single IP injection of liposome-encapsulated rosiglitazone (1 ml/kg; encapsulated in PC_90%_Cholesterol_10%_ liposomes at a concentration of 1.4 mg/ml rosiglitazone) (Liposome Encapsulated Rosiglitazone, **LER10**).
- Group 6 (*n* = 5): daily administration of both rotenone and rosiglitazone for 60 days (**RCG60**).
- Group 7 (*n* = 5): daily rotenone administration for 60 days, and concomitant administration of liposome-encapsulated rosiglitazone (1 ml/kg) every 3 days for 60 days (**LER60**).
- Group 8 (*n* = 5): administration of empty liposomes (1 ml/kg) every 3 days for 60 days (**LIPO60**).

### In vivo imaging: DARC and OCT

A modified Heidelberg HRA-OCT Spectralis (Heidelberg Engineering) was used for OCT imaging [33]. A posterior pole scanning protocol, centred on the rat optic nerve head, was obtained in each treatment group with TruTrack® software (Heidelberg Engineering), to ensure pixel to pixel correspondence over time. Mean retinal thickness was determined using the HEYEX® thickness map analysis (Heidelberg Engineering), before rotenone administration and 10, 20, 40, and 60 days after the start of the treatment regimen. Longitudinal changes in whole retinal thickness were calculated as a percentage against baseline. Individual layer thickness analysis was carried out after masked manual segmentation of the retinal nerve fibre layer (RNFL), inner plexiform layer (IPL), inner nuclear layer (INL), outer plexiform layer (OPL), outer nuclear layer (ONL), and the inner and outer segments (IS/OS) of the photoreceptors.

DARC imaging was conducted in vivo at baseline and day 10, 20, 40, and 60, using a technique described previously [18, 34, 35]. Briefly, the retina was imaged using a confocal scanning laser ophthalmoscope (HRA Spectralis; Heidelberg Engineering) to obtain a baseline image. Next, 2 h after intravitreal administration of fluorescein Isothiocyanate-Annexin A5 (0.25 μg/ml; BD Biosciences), apoptotic RGCs were visualised as bright spots. These were counted manually by three masked observers, deducting baseline autofluorescence spots as described in [34].

### Histological analysis

Rats were sacrificed by CO_2_ asphyxiation, followed by cervical dislocation to ensure death. Upon cardiac perfusion with 4 % paraformaldehyde (PFA) (Sigma-Aldrich), brains were isolated, post-fixated in 4 % paraformaldehyde (Sigma-Aldrich), and 5 μm paraffin-embedded sections were made. For immunohistological detection of tyrosine hydroxylase, sections were rehydrated, endogenous peroxidases were blocked in 3 % H_2_O_2_, and antigen retrieval was performed using citrate buffer (10 mM, pH 6.0) at 90 °C for 15 min. Endogenous proteins were then blocked in 5 % normal donkey serum (DAKO) for 1 h, before antibody incubation was carried out overnight at 4 °C (1:500 anti-tyrosine hydroxylase, MAB318, Millipore). Visualisation was performed using a 3,3′-Diaminobenzidine (DAB) kit (Novolink Polymer Detection System, Leica Biosystems) according to the manufacturer’s instructions and sections were counterstained with Harris haematoxylin (Leica biosystems). Images were acquired with an epifluorescence microscope (BX50; Olympus) and photographed (U-TVIX; Olympus). For quantification of TH positive cells, manual counts of positively stained cells was performed by three masked operators for each section using the “multipoint selection tool” of FIJI software (Fiji Is Just ImageJ, http://fiji.sc/Fiji) [36]. Counts were exported to GraphPad Prism ver. 5.00 for Microsoft Windows (GraphPad Software, San Diego, CA, USA) and the mean was calculated with SEM for statistical analysis.

Optical density of TH immunoreactivity in the striatum was quantified using FIJI software (Fiji Is Just ImageJ, http://fiji.sc/Fiji). Optical density is considered as a reliable method to asses fibres density and to detect changes [37]. Automatic DAB colour deconvolution was used to isolate the TH channel. Striatum area was identified and mean grey pixel intensity recorded [38]. Optical density measures were determined in every striatal section from each rat brain. To measure staining density in the striatum, optical density measures included the entire striatal region of each section [39].

### Statistical analysis

Unless otherwise stated, all values are mean ± Standard Error. Longitudinal comparison of results from OCT and DARC measurements was performed via a one-way ANOVA with repeated measurements by row; all other experimental data were analysed via a one-way ANOVA followed by Tukey’s *post hoc* tests. *P*-values <0.05 were considered significant, with *p < 0.05; **p < 0.01, ***p < 0.005. Significant deviations of retinal layer thickness from baseline values (set at 100 %) were calculated with a one-sample *t*-test, and are indicated with #. All statistical analyses were performed using GraphPad Prism (GraphPad Software).

## Results

### The rotenone-induced model of PD is associated with specific retinal changes over time

**Fig. 1:**
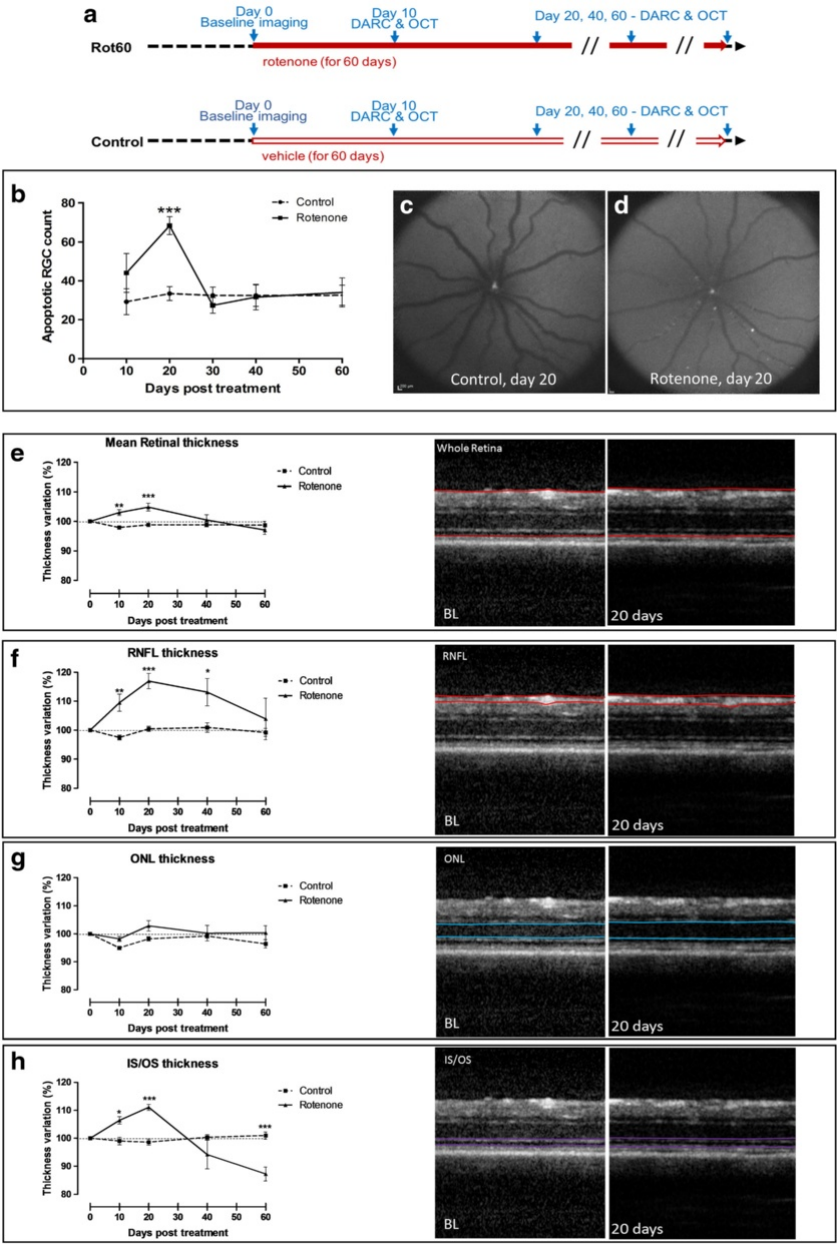
Natural history of rotenone-induced retinal degeneration. **a** Schematic overview of the treatment regimens. **b** Real time in vivo assessment of RGC death by means of DARC reveals a peak in apoptotic cell counts at day 20 of the rotenone treatment scheme. **c**, **d** Representative DARC images of vehicle- (control) and rotenone-treated retinas at day 20. **e**-**h** Morphometric analysis of the thickness of the whole retina, NRFL, ONL, and photoreceptor IS/OS on OCT images indicates a time-dependent characteristic swelling and thinning of the retinal layers in rotenone- versus vehicle-treated rats

In order to characterise the natural history of retinal involvement in the rotenone model, retinal imaging was performed at 10, 20, 30, 40 and 60 days after IP rotenone administration, and showed significant changes in the retina that evolved over time (Fig. 1a-d). Rotenone treated animals exhibited elevated RGC apoptosis compared to vehicle controls which peaked at day 20 (*p* < 0.001) (Fig. 1b-d). No significant difference was found between 30 and 60 days compared to vehicle controls (Fig. 1b).

Whole retinal thickness, as measured by OCT, was significantly increased in rotenone animals compared to vehicle controls at 10 and 20 days (*p* < 0.005; *p* < 0.001), with a maximal difference observed at 20 days of rotenone administration (Fig. 1e). No significant difference between rotenone and control animals was found at any other time point. Segmentation of retinal layers from OCT images revealed a significant increase in RNFL thickness in rotenone-treated eyes compared to control at 10, 20 and 40 days (*p* < 0.005; *p* < 0;001; *p* < 0.05), with peak RNFL thickening at 20 days (Fig. 1f). No significant thickening of the ONL was found over the course of the study (Fig. 1g). However, the thickness of the photoreceptor layer (IS/OS) was significantly increased in the rotenone-treated rats at 10 and 20 days (*p* < 0.05; *p* < 0.001), with a subsequent thinning at 60 days (*p* < 0.001) (Fig. 1h).

### Retinal changes occur before CNS changes in the rotenone-induced PD model

**Fig. 2:**
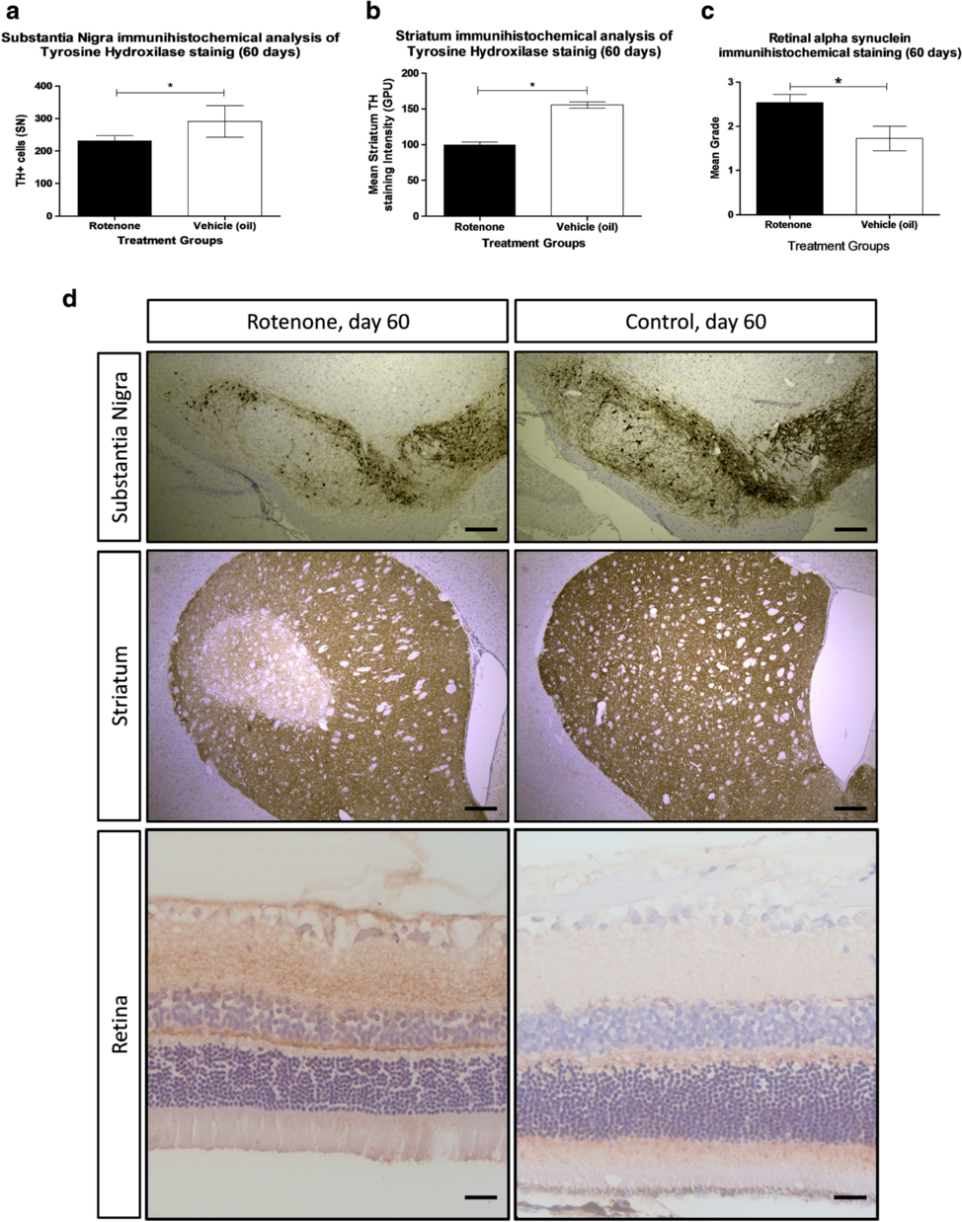
Histological evidence of rotenone-induced neurodegeneration in the SN, striatum and retina. **a** Quantitative grading of TH immunostaining on coronal sections of the SN reveals that significant loss of dopaminergic neurons is seen at day 60 of the rotenone treatment, yet not at day 10 or 20. **b** Significantly reduced immunolabeling is also seen in the striatum of rotenone-treated rats at day 60. **c** Significant increase in Alpha Syinuclein staining was also present in the retina in rotenone treated rats at 60 days. **d** Representative images of TH immunostainings in the SN and striatum, revealing dopaminergic cell loss after 60 days of rotenone treatment alongside retinal histological section revealing increase in alpha synuclein staining in rotenone treated animals. Scale bar, 500 μm

Histological assessment of animal brains was performed in the same animals. As in post mortem specimens from human PD patients, a significant reduction in the number of tyrosine hydroxylase (TH) positive neurons was observed in the substantia nigra (SN) (*p* < 0.05) (Fig. 2a, d) and significant reduction in DAB striatum intensity was observed (*p* < 0.05) (Fig. 2b, d) in rotenone-treated rats at 60 days. No significant change in gross histological TH staining was observed after 10 or 20 days of rotenone administration. Systemic administration of rotenone, including intravenous (i.v.), subcutaneous (s.c.) or intraperitoneal (I.P.) has been found to induce the formation of α-syn cytoplasmatic particles in the substantia nigra of treated rats in a similar manner to typical LB’s found in PD [7, 40]. Our results (data not show) indicate a moderate increase of AS staining in the SN of rats exposed to rotenone for 60 days. However, a significant increase in AS expression in the Rat RGCs 60 days post rotenone administration was found as illustrated in Fig. 2c, d. Taken with the in vivo findings reported above, this highlights the fact that significant changes in the retina occur before the classical PD changes in the brain.

### Rosiglitazone is neuroprotective to retinal cells in vitro

**Fig. 3:**
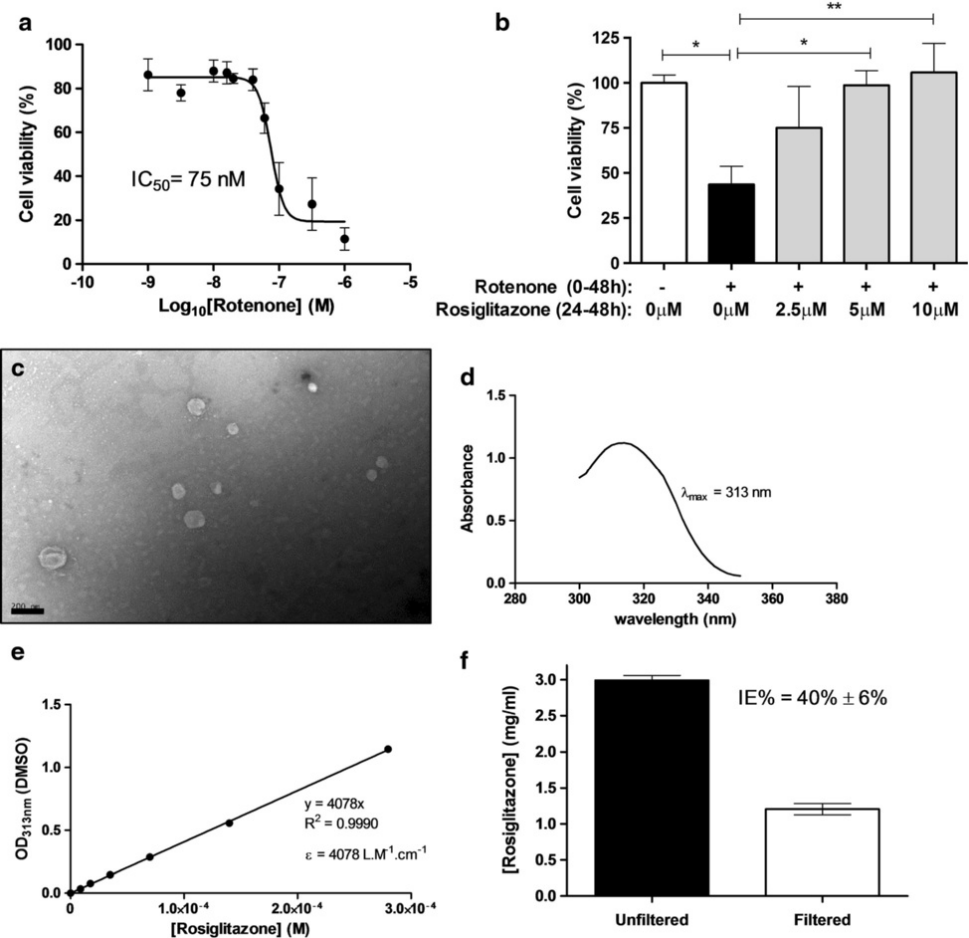
Rosiglitazone treatment protects retinal cells from rotenone-induced toxicity in rat primary mixed cell cultures and characterisation of a liposomal formulation of rosiglitazone. **a** AlamarBlue cell viability assays with mixed rat retinal cell cultures were used to determine the IC50 value of rotenone after 48 h exposure. **b** Rosiglitazone treatment, applied during the second half of the 48 h rotenone insult, conferred dose-dependent neuroprotection to mixed rat retinal cells. Results are representative of three repeated experiments. **c** 1 % Uranyl acetate stained TEM micrograph of rosiglitazone loaded liposomes. Scale Bar = 200 nm. **d** UV-absorbance spectra of rosiglitazone dissolved in DMSO demonstrating peak absorbance at 313 nm. **e** Plot of rosiglitazone concentration in DMSO against absorbance at 313 nm obeys Beer-Lambert’s law up to a concentration of 0.3 mM with a molar extinction coefficient of 4078 L.M-1.cm-1. **f** Incorporation efficiency of rosiglitazone containing liposomes was assessed spectroscopically before and after removal of unencorporated material by filtration. achieving a formulation incorporation efficiency of 40 % ± 6 % (*n* = 5)

The neuroprotective effectiveness of rosiglitazone was first assessed in vitro using mixed, primary rat retinal cell cultures. Primary mixed retinal cultures were found to be susceptible to rotenone-induced cytotoxicity, with an IC_50_ of 75 nM after 48 h of exposure (Fig. 3a). Retinal cultures were subsequently exposed to a 48-h rotenone insult (75 nM) in conjunction with 0 μM, 2.5 μM, 5 μM or 10 μM rosiglitazone treatment for 24 h (between 24 and 48 h of rotenone exposure) dissolved in DMSO. A significant neuroprotective effect, counteracting rotenone-induced cytotoxicity, was observed for 5 μM and 10 μM rosiglitazone (*p* < 0.05; *p* < 0.01) (Fig. 3b).

### Rosiglitazone can be dissolved in a novel liposomal formulation which is clinically translatable

As we have recently shown, due to significant toxicity even at low concentrations, DMSO should be avoided in vivo [30]. The lipophilic nature of rosiglitazone means that it has previously been administered in vivo in oil [41], but due to some drawbacks with the clinical applicability of this methodology, we next attempted to dissolve rosiglitazone in a novel liposomal formulation, which was then characterised (Fig. 3c-f). Liposomal formulations were prepared using the lipid film hydration technique. After 0.22 μm filtration to remove unencapsulated rosiglitazone, formulation size and dispersion were characterised using dynamic light scattering (average Z-diameter 154.2 nm, dispersity 0.267) and transmission electron microscopy (Fig. 3c). Incorporation efficiency (IE) was determined spectroscopically before and after filtration to separate unencapsulated rosiglitazone and found to be 40 % ± 6 % (Fig. 3d-f).

### Rosiglitazone is neuroprotective to the retina in short term studies in rotenone-induced PD model

**Fig. 4:**
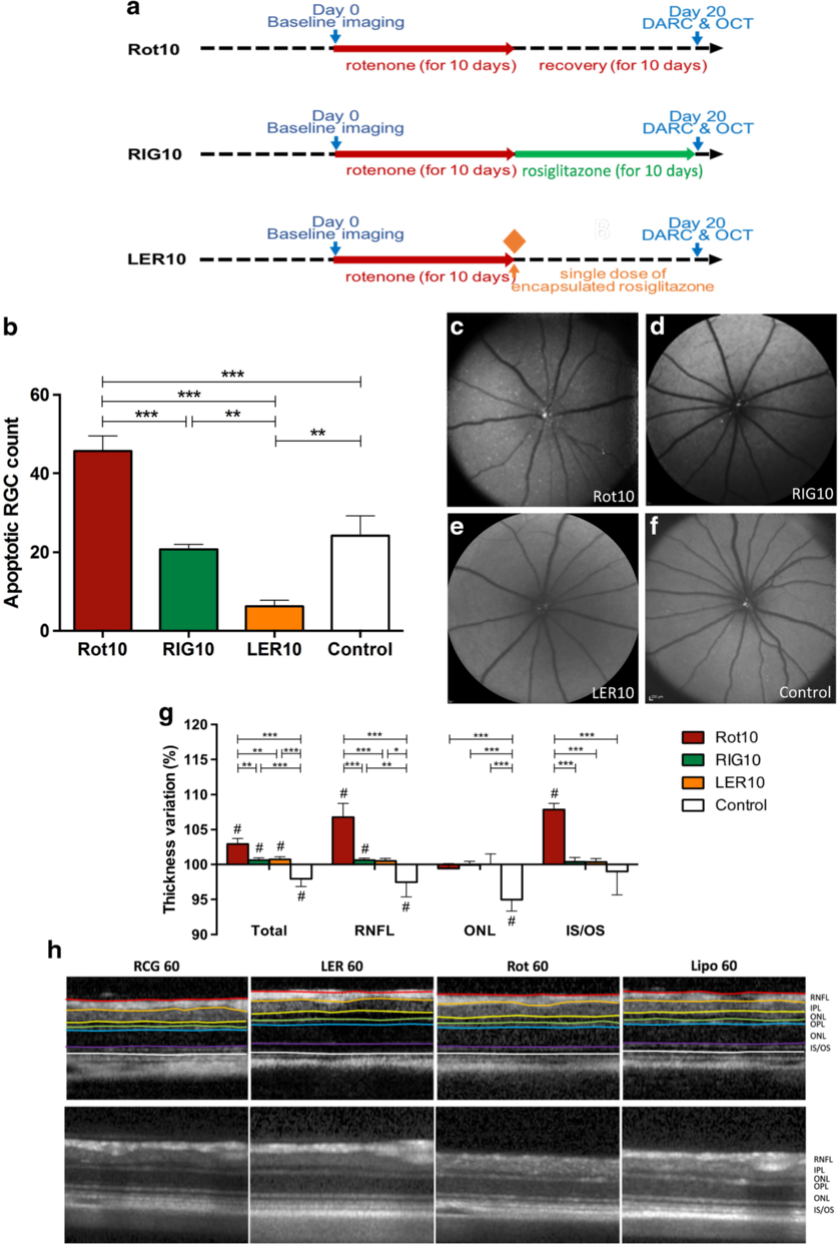
Real time in vivo evidence for a neuroprotective effect of rosiglitazone on retinal neurons. **a** Schematic overview of the treatment regimens. **b** At day 20, DARC imaging reveals significantly reduced counts of apoptotic RGCs in rats that received either 10-days treatment with unencapsulated rosiglitazone (RIG10) or a single administration of liposome-encapsulated rosiglitazone (LER10), compared to rotenone-treated (Rot10) rats. **c**-**f** Representative DARC images for each treatment group. **g** Analysis of retinal thickness variations on OCT images shows that rosiglitazone treatment prevents swelling of the retinal layers at day 20. **h** Representative OCT images for each treatment group

Having established that rosiglitazone is neuroprotective in primary retinal cell cultures, we next evaluated its efficacy in vivo in a short term study of the rotenone induced rodent model of PD. Following a 10-days rotenone insult (Fig. 4a), a significant reduction in RGC apoptosis was observed at day 20 in rats that received a 10-days rosiglitazone treatment (RIG10), compared to those that did not receive therapeutic intervention (Rot10) (*p* < 0.005) (Fig. 4b-d). Furthermore, a liposomal rosiglitazone treated animals (LER10) exhibited a significantly reduced apoptotic RGC count versus those dosed with unencapsulated rosiglitazone (RIG10, *p* < 0.01) or the rotenone vehicle (control, *p* < 0.01) (Fig. 4b, e, f).

Assessment of OCT images confirmed a reduced thickening of the whole retina in RIG10 and LER10 versus Rot10 animals (*p* < 0.01) (Fig. 4g-h). Subsequent analysis of individual layers revealed that rosiglitazone reduced the rotenone induced thickening of the RNFL (*p* < 0.005) and photoreceptor IS/OS (*p* < 0.005) (Fig. 4g-h). Administration of the rotenone vehicle (control) was found to induce a significant thinning of the whole retina, specifically the RNFL and ONL layers (Fig. 4g-h). This observation is in agreement with DARC results, suggesting IP administered glyceryl trioctanoate can induce some retinal toxicity.

### Rosiglitazone effects can be determined in retina before they are seen in the brain

**Fig 5:**
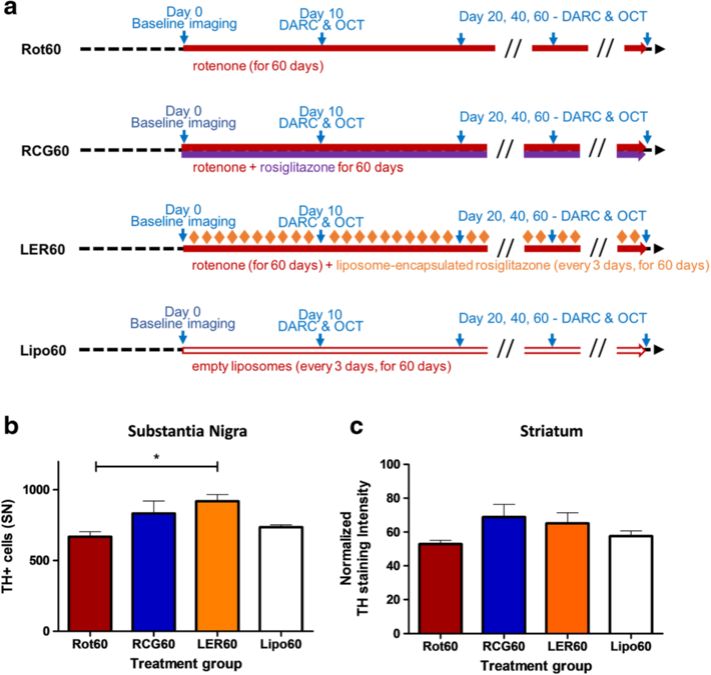
Systemic administration of liposome-encapsulated rosiglitazone (LER) confers neuroprotection to neurons in the retina and in the nigrostriatal pathway. **a** Schematic overview of the treatment regimens. **b** Upon administration of liposome-encapsulated rosiglitazone, every 3 days during the 60-days rotenone treatment (LER60), a neuroprotective effect on tyrosine hydroxylase-positive dopaminergic neurons is seen in the SN. This is not the case when daily injections of unencapsulated rosiglitazone are given (RCG60). **c** A preservative trend of tyrosine hydroxylase-positive efferent fibers in the striatum, in comparison to rotenone only (Rot60), vehicle (empty liposomes) and unencapsulated rosiglitazone (RCG60) rats, is also seen in the striatum

Having established the neuroprotective effects of rosiglitazone therapy in the short term rotenone model of PD, and the suitability of liposomal administration, we next sought to determine whether the therapeutic effects of systemic rosiglitazone observed in the retina at day 20, could predict therapeutic efficacy in the SN and striatum at day 60 (Fig. 5a). Quantification of TH positive cells in the substantia nigra conducted in all four animal groups confirmed a significant reduction in the number of TH positive cells after 60 days of rotenone administration (Fig. 5b). Furthermore, this analysis indicated a significant difference (*p* < 0.05) in the number of TH positive cells between LER60 and Rot60 herby confirming the protective effect of encapsulated rosiglitazone on nigral dopaminergic neurons in this PD model (Fig. 5b). In agreement with results from the short-term model, concomitant administration of unencapsulated rosiglitazone and rotenone for 60 days (RCG60) resulted in a protective effect in the SN which did not achieve significance and was comparable to that achieved on administration of liposomes in the absence of rosiglitazone (Lipo60) (Fig. 5b). Together, these data indicate that systemic administration of liposome rosiglitazone acts to preserve TH positivity in the SN in the rotenone model of PD and to a greater extent than more frequent administration of the unencapsulated drug. Liposomal rosiglitazone therapy (LER60) was found to be more effective at preserving TH positivity in the SN than unencapsulated drug (RCG60) at 60 days (long term model). For the striatum, objective intensity analysis of TH staining of the efferent fibres of the dopaminergic neurons was conducted showing a similar trend compared to the SN however no significant change was seen (Fig. 5c). This result correlates with observations in the retina at 20 days, where liposomal rosiglitazone (LER10) was found to exhibit a greater neuroprotective activity than daily administration of the unencapsulated drug (RIG10) in the retina at 20 days (short term model) using DARC and OCT (*p* < 0.01) (Fig. 4b, g).

## Discussion

These results suggest assessments of retinal health with OCT and DARC can be used to provide reliable indicators of CNS health in a PD model of neurodegenerative disease. Furthermore, the findings suggest that retinal changes can be used as non-invasive and quantitative indicators to predict the efficacy of therapeutic interventions for the treatment of PD.

The treatment of animals with neurotoxins provides important models for the study of the causes and the molecular mechanisms involved in the death of dopaminergic neurons in PD. Although these toxins (e.g. MPTP, paraquat, rotenone) induce PD-like neurodegeneration through different mechanisms, each of them produces different clinical and neuropathological effects, and each is associated with its own advantages and disadvantages, with none perfectly mimicking disease. Similarly, transgenic and viral vector-induced PD models, do not have animals model representing all aspects of human disease. The choice of experimental PD model is related to the specific mechanisms or effect being studied [42]. The rotenone model is known to reproduce most of the motor defect signs and the histopathological features of PD [43, 44] in animals, with human epidemiological studies indicating a correlation between PD and rotenone exposure. Furthermore, rotenone affects the retina, with involvement of non-dopaminergic neurons such as RGCs [45–48]. Finally, additional supporting evidence for the use of a rotenone-induced rat model of PD can be found in epidemiological studies, which indicated a correlation between PD and rotenone exposure in humans [49, 50].

As an extension of the CNS, the retina displays numerous similarities to the brain in terms of cell types, neural circuit organization and molecular signalling pathways. Evidence for a strong association between brain and retinal degeneration can be found in the occurrence of ocular manifestations in many neurodegenerative diseases, including PD [51]. As the underlying mechanisms leading to cell death appear to be conserved between the eye and brain, the retina may provide an early indication of the degeneration elsewhere in the CNS [52].

In this study, DARC imaging was used to demonstrate an increase in RGC apoptosis in the retina of animals subjected to daily systemic rotenone injections. Increased RGC apoptosis was observed at early time points during the study, reaching significance at day 20. In contrast, loss of dopaminergic neurons in the SN and their efferent fibres in the striatum was only observed at day 60. This suggests that elevated RGC apoptosis could be an early feature of PD, in line with findings in animal models of other neurodegenerative diseases including Alzheimer’s disease, glaucoma and Leber’s hereditary optic neuropathy (LHON) [53–55]. In addition, these results confirm previous in vitro and in vivo studies that described a dose-dependent susceptibility of retinal neurons to rotenone-induced cell death [56–58].

OCT analysis showed peak swelling of whole retina, RNFL and photoreceptor IS/OS layers after 20 days of rotenone administration. This was followed by a progressive thinning of the retina between day 20 and 60. The occurrence of peak RGC apoptosis and concomitant retinal swelling at 20 days could be a result of inflammation, as rotenone administration to retinal neuronal cells in vitro is documented to promote the expression of pro-inflammatory JNK and p38 MAPK pathways and PD is inherently linked to neuroinflammatory components [46, 59, 60]. Alternatively, increased mitochondrial biogenesis or axonal transport stasis preceding RGC loss (RNFL thinning) may explain the observed retinal thickening, a phenomenon previously described in the early stages of LHON [61–63], a disease also characterized by mitochondrial complex I dysfunction [54]. The pathological process of swelling and thinning was most prominently observed in the photoreceptor IS/OS, which may be related to the fact that photoreceptors are highly energy-demanding cells bearing a high density of mitochondria [64]. Rotenone administration appears to initially cause ellipsoid swelling, with a subsequent inner segment thinning due to the loss of cellular mitochondria. Other studies have supported these findings by demonstrating neuronal swelling occurring early after rotenone treatment [65], and have also provided histological evidence for substantial inner segment disruption in a rat rotenone model of PD [56]. At present, clinical OCT examination of PD patients has described progressive RNFL thinning as the disease progresses [66–68]. The failure to identify RNFL thickening in PD patients may be a result of the late stage at which the disease is presently diagnosed.

Together, these results suggest that retinal changes occur soon after the onset of disease in the rotenone model of PD and that RGC apoptosis, retinal layer thickness and photoreceptor health could provide early diagnostic markers for PD. Techniques such as DARC and OCT could therefore have early diagnostic potential for PD and provide early and quantitative endpoints for the assessment of neuroprotective strategies.

DARC and OCT imaging technologies were next applied to assess the retinal neuroprotective effects of rosiglitazone. A significant neuroprotective effect of systemic rosiglitazone administration on RGCs and dopaminergic nigrostriatal neurons in the rat model of PD was found. An explanation for the therapeutic effect of rosiglitazone treatment is likely to be due to its PPAR-γ agonist activity, resulting in mitigation of oxidative stress, reduced microglia activation, diminished pro-inflammatory cytokines release, and promotion of mitochondrial biogenesis [25, 69, 70]. These pathways have been implicated in the pathogenesis of PD, suggesting a mechanism of action for the benefit of PPAR-γ agonists in the treatment of early PD reported here and elsewhere [19–26].

In order to enhance the solubility of rosiglitazone, a novel homogeneous liposomal formulation was prepared. Administration of this formulation in the rotenone induced rat model of PD was found to result in a greater neuroprotective effect in both the retina (day 20) and brain (day 60) than daily administration of the unencapsulated drug. The greater efficacy of liposomal rosiglitazone formulations versus administration of the unencapsulated drug could be a result of enhanced drug bioavailability [71, 72]. Several studies have shown great therapeutic potential for liposome incorporated neuroprotective agents, such as NGF, GDNF and minocycline [73–75]. An alternative explanation is a synergistic effect between rosiglitazone and phosphatidylcholine, which has previously been reported to exhibit modest neuroprotective effects in rodent models via anti-oxidant and anti-inflammatory activities [76, 77].

Early and quantitative biomarkers are urgently required for the accurate assessment of therapeutic efficacy in clinical trials for the treatment of neurodegenerative disease [78]. In this study, therapeutic efficacy, assessed using retinal changes with DARC and OCT at day 20, were found to correlate with histological observations in the brain at day 60. For example, administration of a liposomal formulation of rosiglitazone proved to be beneficial in reducing neuronal loss in the retina and brain, while the failure of concomitant rosiglitazone treatment to ameliorate DARC and OCT measures in the retina was a predictor of its lack of therapeutic efficacy in the brain.

## Conclusions

In conclusion, this study provides evidence of the rotenone model as a valuable tool in PD research. We revealed that rotenone induces several structural alterations in the retina and brain, resembling pathological human manifestations, and that the retina can be used as a surrogate marker for changes in the brain. Moreover, the results advocate use of the retina as an endpoint for the evaluation of possible therapeutic strategies in PD. This work highlights the advantages of using real time parameters to evaluate tissue changes in the retina (DARC and OCT) in this PD model, methodologies that due to their non-invasive nature and widespread availability, could easily be applied to the patient. Finally, this study substantiates the evidence for PPAR-γ agonists as promising neuroprotective drugs in PD [79, 80].
